# Escins Isolated from *Aesculus chinensis* Bge. Promote the Autophagic Degradation of Mutant Huntingtin and Inhibit its Induced Apoptosis in HT22 cells

**DOI:** 10.3389/fphar.2020.00116

**Published:** 2020-02-25

**Authors:** Yueshan Sun, Xueqin Jiang, Rong Pan, Xiaogang Zhou, Dalian Qin, Rui Xiong, Yiling Wang, Wenqiao Qiu, Anguo Wu, Jianming Wu

**Affiliations:** ^1^School of Pharmacy, Southwest Medical University, Luzhou, China; ^2^The Key Laboratory of Medical Electrophysiology, Sichuan Key Medical Laboratory of New Drug Discovery and Druggability Evaluation, Luzhou Key Laboratory of Activity Screening and Druggability Evaluation for Chinese Materia Medica, Southwest Medical University, Luzhou, China; ^3^Department of Pharmacy, Affiliated Hospital of Southwest Medical University, Luzhou, China

**Keywords:** Huntington’s disease, autophagy, mHtt, *Aesculus chinensis* Bge., HT22 cells

## Abstract

The pathogenesis of Huntington’s disease (HD), an inherited progressive neurodegenerative disease, is highly associated with the cytotoxicity-inducing mutant huntingtin (mHtt) protein. Emerging evidence indicates that autophagy plays a pivotal role in degrading aggregated proteins such as mHtt to enhance neuronal viability. In this study, by employing preparative high-performance liquid chromatography (pre-HPLC), ultra-high performance liquid chromatography diode-array-detector quadrupole time-of-flight mass spectrometry (UHPLC-DAD-Q-TOF-MS) and nuclear magnetic resonance (NMR), three escins, escin IA (EA), escin IB (EB) and isoescin IA (IEA), were isolated and identified from the seed of *Aesculus chinensis* Bge. (ACB). After EGFP-HTT74-overexpressing HT22 cells were treated with EA, EB and IEA at safe concentrations, the clearance of mHtt and mHtt-induced apoptosis were investigated by Western blot, immunofluorescence microscopy and flow cytometry methods. In addition, the autophagy induced by these escins in HT22 cells was monitored by detecting GFP-LC3 puncta, P62 and LC3 protein expression. The results showed that EA, EB and IEA could significantly decrease mHtt levels and inhibit its induced apoptosis in HT22 cells. In addition, these three saponins induced autophagic flux by increasing the ratio of RFP-LC3 to GFP-LC3, and by decreasing P62 expression. Among the tested escins, EB displayed the best autophagy induction, which was regulated *via* both the mTOR and ERK signaling pathways. Furthermore, the degradation of mHtt and the commensurate decrease in its cytotoxic effects by EA, EB and IEA were demonstrated to be closely associated with autophagy induction, which depended on ATG7. In conclusion, we are the first to report that the escins, including EA, EB and IEA are novel autophagy inducers that degrade mHtt and inhibit mHtt-induced apoptosis *in vitro* and *in vivo*. As a result of these findings, the triterpenoid saponins in ACB might be considered to be promising candidates for the treatment of HD in the future.

## Introduction

Huntington’s disease (HD), a neurodegenerative disease characterized by progressive motor and cognitive dysfunction, is caused by the mutant huntingtin (mHtt) protein, which is encoded by the huntingtin (HTT) gene that has an expanded CAG repeat (> 36) in chromosome 4 (Dayalu and Albin, 2015; Li et al., 2015; Manoharan et al., 2016; Southwell et al., 2017). In transgenic mice expressing N-terminal mHtt and the HD phenotype, abundant mHtt aggregates were found in neurons of the brain. In addition, the HD knock-in mice also expressed full-length mHtt (Zhao et al., 2016). Emerging evidence has shown that the mHtt aggregates induce cytotoxicity, which is closely related to neuronal death in HD (Lu and Palacino, 2013; Xi et al., 2016), and the reduction in mHtt aggregates has been proven to rescue HD-related phenotypes through genetic and chemical modifications (Jimenez-Sanchez et al., 2015; Yu et al., 2017). Therefore, the clearance of mHtt has become a promising strategy for HD therapy.

Autophagy plays an important role in maintaining cellular homeostasis by degrading damaged or unnecessary materials in cells (Mizushima and Komatsu, 2011; Gitler et al., 2017; Yu et al., 2017; Pandey and Rajamma, 2018). Intracytoplasmic aggregate-prone proteins such as β-amyloid (Aβ), hyperphosphorylated tau, mutant α-synuclein and huntingtin rapidly accumulate as autophagy is impaired in cellular and animal models (Kampmann, 2017; Wu et al., 2018). Additionally, the accumulation of these aggregated proteins is highly associated with their cytotoxic effects in neurons. Therefore, it is essential to recovery the neuronal viability through degrading these toxic aggregated proteins by the autophagy enhancers. Tetrabenazine, a dopamine-depleting agent, is the most efficient drug currently used for the treatment of HD. However, it causes many adverse effects, such as significant depression, lethargy and Parkinsonian syndrome occurrence. Recently, many compounds and natural products have been reported to degrade mHtt *via* autophagy (Dayalu and Albin, 2015; Wyant et al., 2017; Pandey and Rajamma, 2018). For example, rilmenidine, a U.S. Food and Drug Administration (FDA) approved drug, was reported to induce autophagy and attenuate the cytotoxicity induced by mHtt. Trehalose, a natural product, can promote the degradation of α-synuclein and mHtt *via* mTOR-independent pathway (Sarkar et al., 2007; Mercer et al., 2017). Furthermore, we have previously identified components in traditional Chinese medicines (TCMs), such as saponins from *Radix polygala* and *Hedera helix*, and neferine from *Nelumbo nucifera* Gaertn, could induce autophagy *via* AMPK/mTOR pathways to accelerate the degradation of mHtt and inhibit its cytotoxic effects in PC-12 cells (Wu et al., 2013; Vincent et al., 2015; Wu et al., 2017). However, there are still few effective autophagic inducers used in the clinic for the treatment of HD. Therefore, identification of additional potent autophagy inducers that clear mHtt with fewer side effects is urgently needed.

TCMs have been used in China for more than 2000 years, and most of them have been proven to be effective and safe (Jiang et al., 2016). *Aesculus chinensis* Bge. (ACB), a commonly used TCM, was reported to have anti-inflammatory, anti-oedema, anti-cancer, anti-hyperglycaemic, anti-obesity, and anti-ulcerative effects (Sirtori, 2001; Zhang et al., 2010; Zhu et al., 2017). Emerging evidences have indicated that triterpenoid saponins named escins are the main components in ACB (Zhang et al., 2010; Seweryn et al., 2014; Cheng et al., 2016) and were reported to exhibit neuroprotective effect *via* both anti-oxidative and anti-apoptotic action mechanisms in a chronically MPTP/p-induced PD mouse model (Selvakumar et al., 2015). Moreover, escins also reduced oxidative stress and attenuated cognitive deficits and hippocampal injury in mice with transient global cerebral ischaemia (Cheng et al., 2016). However, little research on their ability to induce autophagy and/or degrade aggregated proteins, such as mHtt, has been reported. In this study, by using HT22 cells transiently transfected with EGFP-HTT74 as a HD cellular model, we were the first to find that escins and single saponins, including escin IA (EA), escin IB (EB) and isoescin IA (IEA), from ACB seeds significantly inhibited mHtt protein levels and mHtt-induced apoptosis. The degradation effect of mHtt is closely linked with autophagy induction, which is regulated *via* both the mTOR and ERK signaling pathways. Therefore, the findings in this study offer important novel insights that may enable further development of the triterpenoid saponins from ACB as autophagy enhancers into novel neuroprotective agents.

## Materials and Methods

### Materials and Reagents

SCH772984 (SCH, T6066) and 3-methyladenine (3MA, T1879) were purchased from Topscience Co., Ltd. (Shanghai, China). Bafilomycin (Baf, B101389) was purchased from Aladdin Bio-Chem Technology Co., Ltd. (Shanghai, China). Compound C (CC) was obtained from Calbiochem (San Diego, CA, USA). The EGFP-HTT74 plasmid was a generous gift from David C. Rubinsztein (University of Cambridge, Cambridge, UK). The pEGFP-LC3 and mRFP-GFP tandem fluorescent-tagged LC3 (tfLC3) reporter plasmids were kindly provided by Tamotsu Yoshimori (Osaka University, Osaka, Japan). Exfect transfection reagent (T101-01) was obtained from Vazyme Biotech Co., Ltd. (Nanjing, China). A cell counting kit-8 (CCK-8) was purchased from Dojindo Laboratories (Kumamoto, Japan). Antibodies used in this study including p-PI3 kinase (p-PI3K, 4228), PI3 kinase (PI3K, 4249), p-Akt (4060), Akt (4691), p-mTOR (5536), Bax (14796), Bcl-2 (3498), GFP (2955), p-AMPK (2535), p-ULK1 (6888), p-MEK1/2 (9154), MEK1/2 (4694), p-ERK (4370), ERK (4695), p-Raf (2696), Raf (14814), p-P70S6K (9205), β-actin (4970), and anti-rabbit IgG (4414), were obtained from Cell Signaling Technology (Danvers, MA, USA). LC3B (PM036), caspase 3 (M097), and caspase 9 (M054) were purchased from MBL (Nagoya, Japan). Anti-P70S6K (14485-1-AP) was obtained from Proteinch (Wuhan, China). Huntingtin (sc-47757) and ULK1 (sc-390904) came from Santa Cruz Biotechnology Co., Ltd. (Dallas, Texas, USA). AMPK (SH030221E), and mTOR (SA080117AH) were obtained from ABGENT (San Diego, California, USA). Lactate dehydrogenase (LDH) cytotoxicity assay kit was purchased from Beyotime Biotechnology Co., Ltd. (Beijing, China).

### Preparation of ES, EA, EB, and IEA

Air-dried ACB (2 kg) was smashed into crude powder and extracted with methanol for 1 h using an ultrasonic method, which was repeated two times. All the extracted solutions were combined, filtered, and concentrated by a rotary evaporator under vacuum. The dried extract was resuspended in water and mixed with macroporous resin, which was then washed out using 4–6 times 50–70% methanol. The eluted solution was collected and concentrated by rotary evaporator under vacuum to 1/10 times as its original volume and remained undisturbed to crystallize. After filtration, the crude crystals obtained were further crystallized for 2–3 times, and the refined crystals (235 mg) obtained were ES. For the additional isolation of EA (34.2 mg), EB (26.4 mg) and IEA (13.6 mg), ES was re-dissolved in methanol and then subjected to separation by preparative high-performance liquid chromatography (pre-HPLC). A total of three main peaks were collected and concentrated for further confirmation by mass spectrometry (MS) and nuclear magnetic resonance (NMR).

### Chromatography Parameters

Analyses of all the samples, including the ACB total extract, ES, EA, EB and IEA, were carried out on a Shimadzu system (Kyoto, Japan) equipped with an LC-3AD solvent delivery system, a SIL-30ACXR auto-sampler, a CTO-30AC column oven, a DGU-20A3 degasser, and a CBM-20A controller. All samples were separated on an Agilent Zorbax Eclipse Plus C_18_ column (100 mm×2.1 mm, 1.8 µm, flow rate: 0.35 mL/min). The column oven temperature was set at 40°C. The mobile phase consisted of A (0.1% formic acid in water) and B (0.1% formic acid in acetonitrile): 0–8 min, 5–70% B; 8–11 min, 70–100% B; 11–12 min, 100% B; 12–15 min, 5% B.

The UHPLC-QTOF-MS/MS analysis was conducted on a triple TOF™ X500R system with a Duo Spray source in the negative electrospray ion mode (AB SCIEX, Foster City, CA, USA). The electrospray ionization was applied in the negative mode with the following parameters: ion spray voltage, –4,500 V; ion source temperature, 500°C; curtain gas, 25 psi; nebulizer gas (GS 1), 50 psi; heater gas (GS 2), 50 psi; and declustering potential (DP), –100 V. The mass ranges were set at *m/z* 60 –2,000 Da for the TOF-MS scan. The LC-MS/MS data were analyzed using the Peak View^®^ 1.4 software (AB SCIEX Foster City, CA, USA).

### Cell Culture

The mouse hippocampal neuron cell line HT22 was purchased from the American Type Culture Collection (ATCC; Rockville, MD, USA). Stable RFP-GFP-LC3 U87 cells were kindly provided by Dr. Xiaoming Zhu in Macau University of Science and Technology. The wild-type ATG7 (ATG7^+/+^) and ATG7-deficient (ATG7^–/–^) mouse embryonic fibroblasts (MEF) were provided by Masaaki Komatsu (Juntendo University, School of Medicine, Tokyo, Japan). All cells were cultured in DMEM (except for U87 in MEM-Alpha) supplemented with 10% FBS, penicillin (100 U/mL), and streptomycin (100 μg/mL) at a 37°C; humidified incubator with 5% CO_2_ atmosphere.

### Cell Viability and Cytotoxicity Assay

The cell viability of HT22 cells upon the treatment with ES, EA, EB and IEA as indicated concentrations was detected by CCK-8 assay according to the manufacturer’s protocol. In brief, the cells were seeded in 96-well plates at a density of 5×10^3^ cells/well overnight and then treated with ES (1.5625–200 μg/mL), EA, EB or IEA (1.25–80 μM) for 24 h, respectively. After treatment, the medium was replaced by 100 μL of DMEM with 10% CCK-8 solution and followed by an incubation for 1 h. The absorbance value of the solution was measured at 450 nm using a Thermo Scientific Microplate Reader. The percentage of cell viability was calculated using the following formula: cell viability (%) = cell number treated/cell number DMSO control ×100. Data were obtained from three independent experiments.

The cell viability was also measured by flow cytometry using an annexin V-FITC apoptosis staining/detection kit (4A Biotech Co., Ltd, Beijing, China). After treatment, HT22 cells were trypsinzed and incubated with 1× Annexin V solution containing FITC and PE reagents for 15 min according to the manufacturer’s instructions. Flow cytometry analysis was then carried out by using a FACSVerse flow cytometer (BD Biosciences, New Jersey, USA). Data analysis was performed using FlowJo 7.6 software (FlowJo LLC., Oregon, USA). The data were obtained from three independent experiments.

The cytotoxicity of ES, EA, EB and IEA against HT22 cells was assessed by determining the release of LDH from cells using a LDH cytotoxicity assay kit according to the manufacturer’s protocol. In brief, HT22 cells were seeded in 96-well plate and treated with ES, EA, EB, and IEA as indicated concentrations for 24 h. After treatment, the supernatant was collected and the LDH activity was measured by using a Thermo Scientific Microplate Reader at 490 nm. The cytotoxicity was obtained by calculating relative percentage of LDH release according to the formula: LDH release (%) = (OD_treated_–OD_control_)/(OD_max_–OD_control_) ×100. All experiments were performed at least three times in triplicate.

### Quantification of GFP-LC3 Puncta Formation

To detect the induction of autophagy, cell suspensions of HT22 or MEFs at a density of 2.5×10^5^ cells/well were seeded onto the coverslips in 6-well plates. After 12 h, the cells were transfected with pEGFP-LC3 for another 12 h using liposome-mediated transfection reagent (T101-01, Vazyme Biotech), followed by the indicated treatments for another 24 h. After treatments, the cells were washed with PBS and fixed with 4% paraformaldehyde (PFA) at room temperature for 15 min. The coverslips were then washed three times with PBS and taken out for air-drying and mounted with FluorSave™ mounting media (Calbiochem, San Diego, CA, USA) for fluorescence microscopic analysis. Representative images were captured with an Olympus microscope. The number of GFP-positive cells and cells with GFP-LC3 puncta formation were counted. The induction of autophagy was quantified by comparing the number of cells showing GFP-LC3 fluorescent puncta to the number of GFP-positive cells to calculate a percentage. Alternately, we also quantified autophagy induction effect by counting the number of the GFP-LC3 puncta in cells. A minimum of 300 cells from 3 randomly selected fields was scored and the count was double-blinded.

### mRFP-GFP Tandem Fluorescent-Tagged LC3 (tfLC3) Detection

HT22 cells seeded on coverslips in 6-well plates were transiently transfected with tfLC3 plasmid for 18–24 h, then followed with a 24 h treatment of EA, EB, IEA, or Rap as indicated concentrations. After treatment, cells were fixed with 4% PFA and mounted by FluorSave™ mounting media. The autophagy effect was examined under the fluorescence microscope with 40× magnification. Representative images of GFP-LC3 and RFP-LC3 at the same filed were captured and merged for determining the autophagy flux by calculating the GFP/RFP fluorescence ratio using ImageJ software (ImageJ 1.46r; National Institutes of Health, Bethesda, MD, USA) (Zhou et al., 2012; Wan et al., 2018). The intensities of GFP and RFP signals were from 3 randomly selected fields.

### Western Blot Analysis

After drug treatment, the cells were harvested and lysed with 1× RIPA lysis buffer (Cell Signaling Technology, Beverly, MA, USA) containing protease inhibitor cocktail with EDTA-free (TargetMol, Shanghai, China). The cell lysates were then centrifuged at 12,000 rpm for 10 min at 4°C. The supernatant was transferred to a 1.5 mL tube, and the protein concentration was quantified by using the Quick Start™ Bradford 1× Dye Reagent (Bio-Rad, Hercules, CA, USA). Thirty micrograms of protein from each sample were loaded on SDS-PAGE gel and separated at 120 V for 90 min. After electrophoresis, the proteins were transferred onto a PVDF membrane (Millipore, Darmstadt, Germany) that was then blocked with 10% non-fat dried milk at room temperature for 1 h. The membranes were then incubated with the primary antibodies at 4°C overnight, followed by an incubation with HRP-conjugated secondary antibody for 1 h at room temperature. The bands were revealed using UltraSignal™ ECL Western blot detection reagents (4A Biotech Co., Ltd, Beijing, China) and detected by a ChemiDoc MP Imaging System (Bio-Rad). The band intensity was quantified by using ImageJ software (ImageJ 1.46r; National Institutes of Health, Bethesda, MD, USA), and the ratio of the protein of interest to β-actin was calculated. The data were obtained from three independent experiments.

### Detection of Mutant Huntingtin Protein and Inclusions

The HT22 or MEF cells were transfected with EGFP-HTT74 plasmid for 12 h using Exfect transfection reagent (Vazyme Biotech Co., Ltd, Nanjing, China) and then treated with the indicated drugs for 24 h. After treatment, the cells were collected for the detection of HTT levels using immunoblotting with an antibody against GFP or huntingtin. Additionally, GFP was also quantified by detecting the GFP signal using flow cytometry. For the fluorescence microscopy analysis of cytoplasmic huntingtin inclusion formation, the cells grown on the coverslips were observed under an Olympus fluorescence microscope. The quantification of EGFP-HTT74 inclusion was performed by calculating the percentage of the number of cells with EGFP-HTT74 inclusions to the total number of EGFP-positive cells. Alternately, the quantification of EGFP-HTT74 inclusion was also obtained by counting the number of EGFP-HTT74 inclusion in single cell (Rossi et al., 2009; Zhou et al., 2012). A minimum of 300 GFP-positive cells from 3 randomly selected fields was scored and the count was double-blinded.

### Statistical Analysis

Statistical significance among the groups was analyzed by one-way univariate analysis of variance (ANOVA) using GraphPad Prism 5.0 software (GraphPad Software, Inc., La Jolla, CA, USA). *P<0.05, **P<0.01 and ***P<0.001 were considered to be significant.

## Results

### The Identification of ES, EA, EB, and IEA

**Figure 1:**
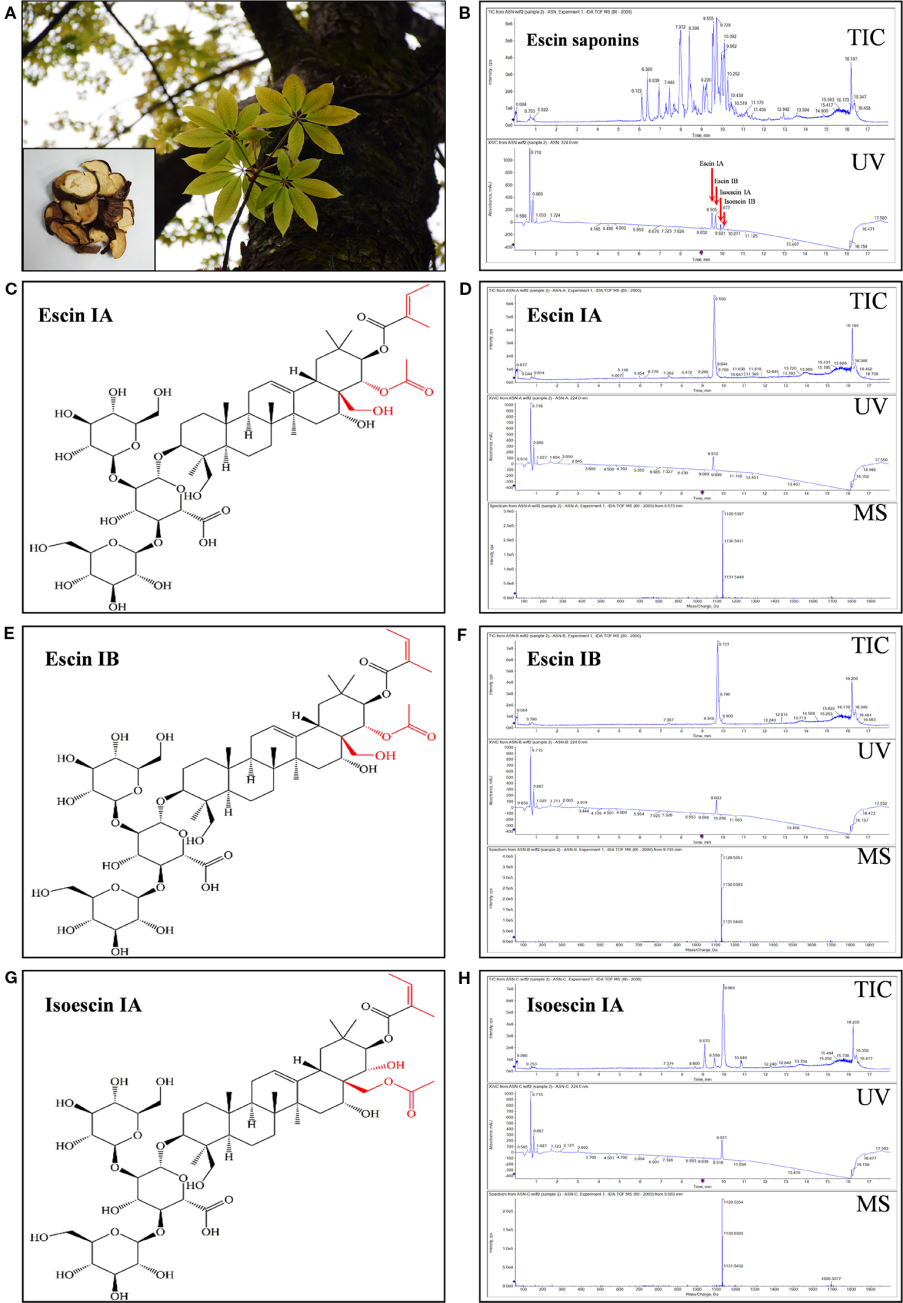
Isolation and identification of ES, EA, EB and IEA from the seeds of *Aesculus chinensis* Bge. **(A)** Photos of the tree of *Aesculus chinensis* Bge. and its sliced seed. **(B)** Total ion chromatogram (TIC) and diode array detector (DAD) chromatogram with UV at 224 nm for the purified total saponin named escins (ES). **(C)** The structure of EA. **(D)** The TIC and DAD chromatogram with UV set at 224 nm for EA. **(E)** The structure of EB. **(F)** The TIC and DAD chromatogram with UV set at 224 nm for EB. **(G)** The structure of IEA. **(H)** The TIC and DAD chromatogram with UV set at 224 nm for IEA. All chromatograms were obtained using a Shimadzu liquid system equipped with a triple TOF™ X500R system in negative electrospray ion mode.

The total saponin named ES and single saponin, such as EA, EB, or IEA, used in this study were isolated from ACB seeds by us (**Figure 1A**). After the seed of ACB was extracted using ultrasonic method with methanol, the total ACB extract was analyzed by UHPLC-QTOF-MS/MS. As shown in **Figure S1**, the total ion chromatogram (TIC) and UV chromatogram display that saponins, such as EA, EB, IEA and isoescin IB (IEB) in ACB, are the peaks between 9 and 11 min. The total saponin in the ACB extract were then isolated and purified by chromatography with a macroporous resin column and the eluted solution was used for crystallization, then ES was obtained. After analysis by using UHPLC-QTOF-MS/MS, EA, EB, IEA and IEB were found with a high proportion in ES (**Figure 1B**). For further isolation of the single saponin in ES, a total of three compounds, EA, EB and IEA, were successfully isolated, and their responses to total ion chromatography (TIC) and UV detected chromatograms are shown in **Figures 1D, F, H**. Their molecular formulas were determined to be C_55_H_86_O_24_ based on their HR-ESI-MS *m/z* values: 1129.5367 [M–H]^–^, 1,129.5351 [M–H]^–^ and 1,129.5354 [M–H]^–^ in negative mode, respectively. The ^1^H NMR (CDCl_3_, 600 MHz) and ^13^C NMR (CDCl_3_, 150 MHz) spectra of the compounds are shown in **Figures S2**–**S7**. These NMR data are consistent with the reported compounds in the literatures (Chen et al., 2007; Wu et al., 2012b). Therefore, these compounds were confirmed as EA, EB, and IEA. Their respective structures are shown in **Figures 1C, E, G**, and their differences in structure are labeled in red.

### Safe Concentrations of ES, EA, EB, and IEA in HT22 Cells

**Figure 2:**
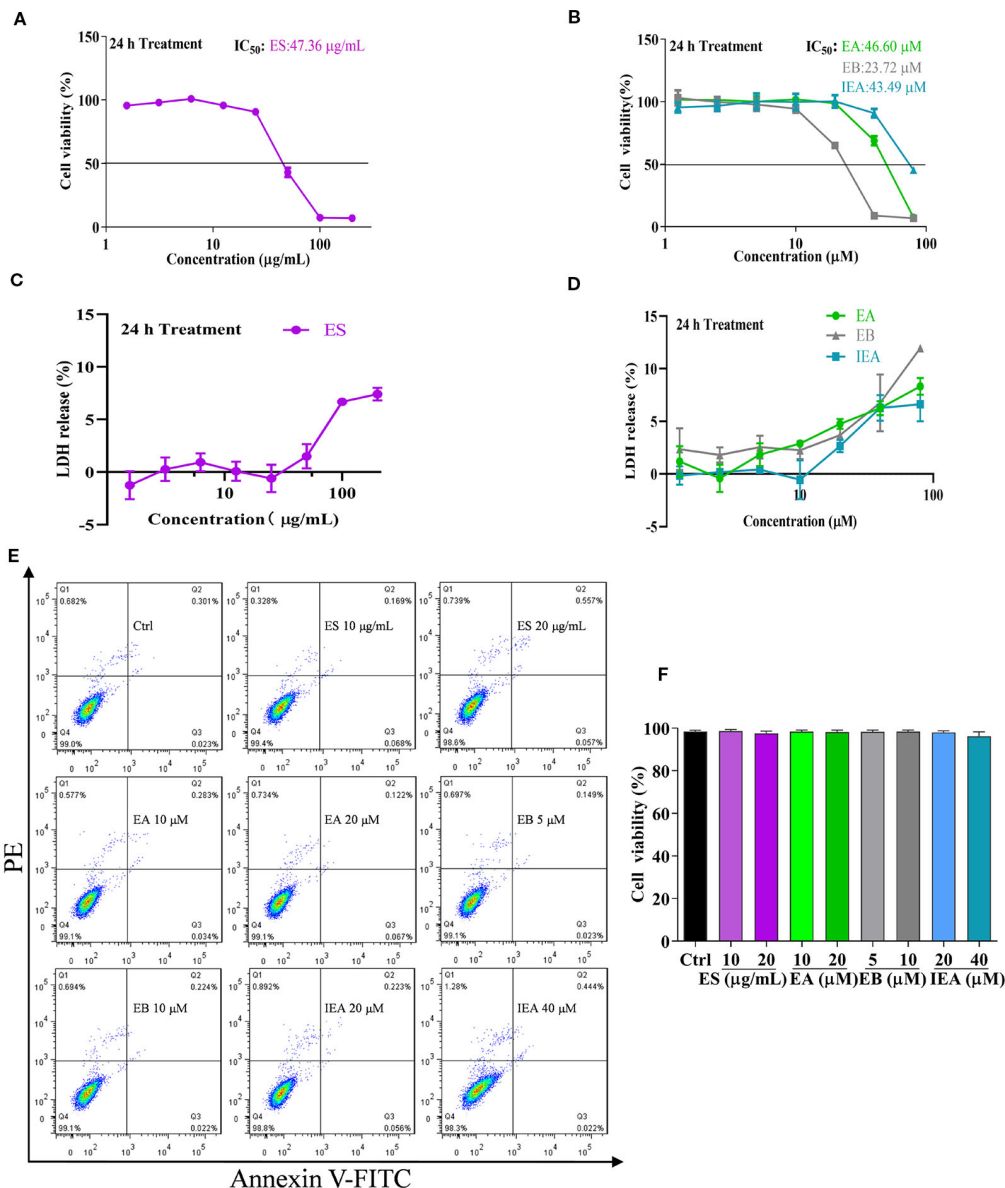
Safe concentrations of ES, EA, EB and IEA in HT22 cells. **(A, B)** HT22 cells were treated with ES (1.5625, 3.125, 6.25, 12.5, 25, 50, 100, and 200 μg/mL) or EA, EB and IEA (1.25, 2.5, 5, 10, 20, 40, and 80 μM) for 24 h, the cell viability was then detected by using a CCK-8 assay kit. **(C, D)** HT22 cells were treated with ES (1.5625, 3.125, 6.25, 12.5, 25, 50, 100, and 200 μg/mL) or EA, EB, and IEA (1.25, 2.5, 5, 10, 20, 40, and 80 μM) for 24 h, the LDH release was then assayed by a LDH cytotoxicity detection kit. **(E)** HT22 cells were treated with ES, EA, EB, and IEA at the indicated concentrations for 24 h, the cell viability was then analyzed by flow cytometry using an annexin V-FITC/PI kit. **(F)** Bar chart indicates the cell viability of HT22 cells that analyzed by flow cytometry.

Before investigating the degradation effect of mHtt, the safe concentrations of ES, EA, EB, and IEA in HT22 cells were measured by CCK-8 assay. As shown in **Figures 2A, B**, the HT22 cells were treated with 1.56–200 μg/mL ES and 1.25–80 μM EA, EB, and IEA for 24 h. Their IC_50_ values against HT22 cells were 47.36 μg/mL, 46.60 μM, 23.72 μM, and 43.49 μM for ES, EA, EB, and IEA, respectively. Furthermore, the cytotoxicity of ES, EA, EB, and IEA against HT22 cells were measured using LDH cytotoxicity detection kit. The results revealed that there is only less than 15% of cytotoxicity detected in HT22 cells treated with ES, EA, EB, or IEA treated at the maximum concentration (**Figures 2C, D**). Moreover, by using an annexin V-FITC/PI apoptosis detection kit, the cell viability of the HT22 cells treated with ES, EA, EB, and IEA at the indicated concentrations was determined. As shown in **Figure 2E, F**, there was no obvious cytotoxicity observed. Taken together, these results suggest that the safe concentrations of ES, EA, EB, and IEA are lower than 40 μg/mL, 20 μM, 10 μM, and 40 μM, respectively.

### EA, EB, and IEA Reduce the Expression of HTT74 in HT22 Cells

**Figure 3:**
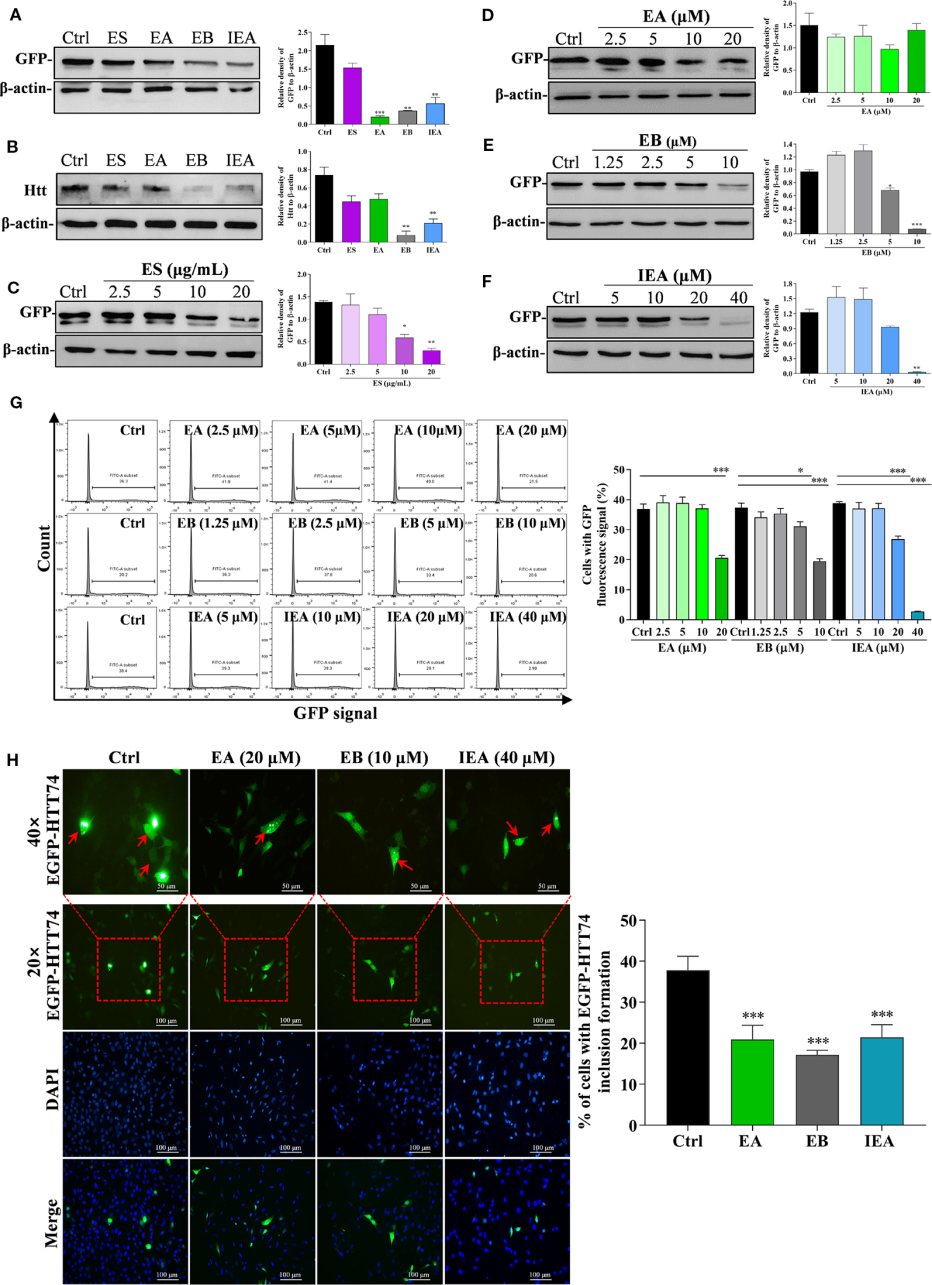
ES, EA, EB, and IEA enhance the clearance of mHtt in HT22 cells. HT22 cells transfected with EGFP-HTT74 were treated with ES (40 μg/mL), EA (20 μM), EB (10 μM), and IEA (40 μM) for 24 h, the protein level of GFP **(A)** and mHtt protein **(B)** was then detected by using Western blot. HT22 cells transfected with EGFP-HTT74 were treated with ES (2.5, 5, 10, and 20 μg/mL) for 24 h **(C)**, EA (2.5, 5, 10, and 20 μM) for 24 h **(D)**, EB (1.25, 2.5, 5, and 10 μM) for 24 h **(E)**, or IEA (5, 10, 20, and 40 μM) for 24 h **(F)**. After treatment, the cells were harvested and the level of mHtt protein was detected by using Western blot with GFP antibody. The bar chart indicates the relative density of GFP or Htt to β-actin; bars, S.D. *P ≤ 0.05, **P ≤ 0.01, ***P ≤ 0.001. **(G)** HT22 cells transfected with EGFP-HTT74 were treated with EA, EB and IEA at the indicated concentrations for 24 h. Cells were then collected and analyzed by flow cytometry using the FITC channel. Bar chart indicates the percentage of cells with GFP signal; bars, S.D. *P ≤ 0.05, ***P ≤ 0.001. **(H)** HT22 cells transfected with EGFP-HTT74 were treated with EA, EB and IEA at the indicated concentration for 24 h. After treatment, the cells were fixed with 4% PFA and representative images were captured by using a fluorescence microscope (Nicon; Japan). Red arrows indicate the cells with formed EGFP-HTT74 inclusion. Magnification: 20× or 40×. Scale bar: 100 μm. Bar chart indicates the percentage of cells with EGFP-HTT74 inclusion formation; bars, S.D. ***P ≤ 0.001. The full-length Western blot images are showed in **Figure S12**.

Huntington’s disease (HD) is an autosomal dominant, inherited neurodegenerative disease, which is mainly due to the excessive expansion of CAG trinucleotide repeats in the huntingtin gene in chromosome 4, leading to the abnormal of polyglutamine near the N-terminal of the HTT (Mizushima and Komatsu, 2011; Dayalu and Albin, 2015; Huang et al., 2018). In addition, the formation of neuronal mHtt aggregates is a pathological marker of HD. mHtt can form perinuclear cytoplasmic aggregates and intranuclear inclusions cause neuronal death. Accordingly, we transiently transfected the EGFP-HTT74 plasmid into HT22 cells to establish a cellular model of HD and evaluated the degradation effect of ES, EA, EB, and IEA on EGFP-HTT74 protein. As shown in **Figures 3A, B**, ES (40 μg/mL), EA (20 μM), EB (10 μM), and IEA (40 μM) significantly inhibited the protein level of mHTT, which was detected by using Western blot with antibodies against GFP and huntingtin. Then, we investigated the concentration-dependent effects of ES, EA, EB, and IEA, the **Figures 3C–F** showed that ES, EA, EB, and IEA could remarkably inhibit the protein expression of GFP. Among these saponins, EB showed the strongest degradation effect compared to equal concentration of other saponins. In addition, the relative high transfection efficiency of EGFP-HTT74 in HT22 cells was firstly confirmed by flow cytometry (**Figure S8**), then the result showed in **Figure 3G** indicated that EA, EB, and IEA could decrease the percentage of cells with GFP signal in EGFP-HTT74 overexpressed HT22 cells. Furthermore, the cytoplasmic EGFP-HTT74 fluorescent inclusions in HT22 cells, visualized using immunofluorescence microscopy, are presented in **Figure 3H**, which showed that EA, EB, and IEA could significantly decrease the EGFP-HTT74 inclusions in HT22 cells. Taken together, these results suggest that saponins, such as EA, EB, and IEA, are the main bioactive components in ACB that facilitate the degradation of mHTT associated with HD.

### EA, EB, and IEA Induce Autophagy in HT22 Cells

**Figure 4:**
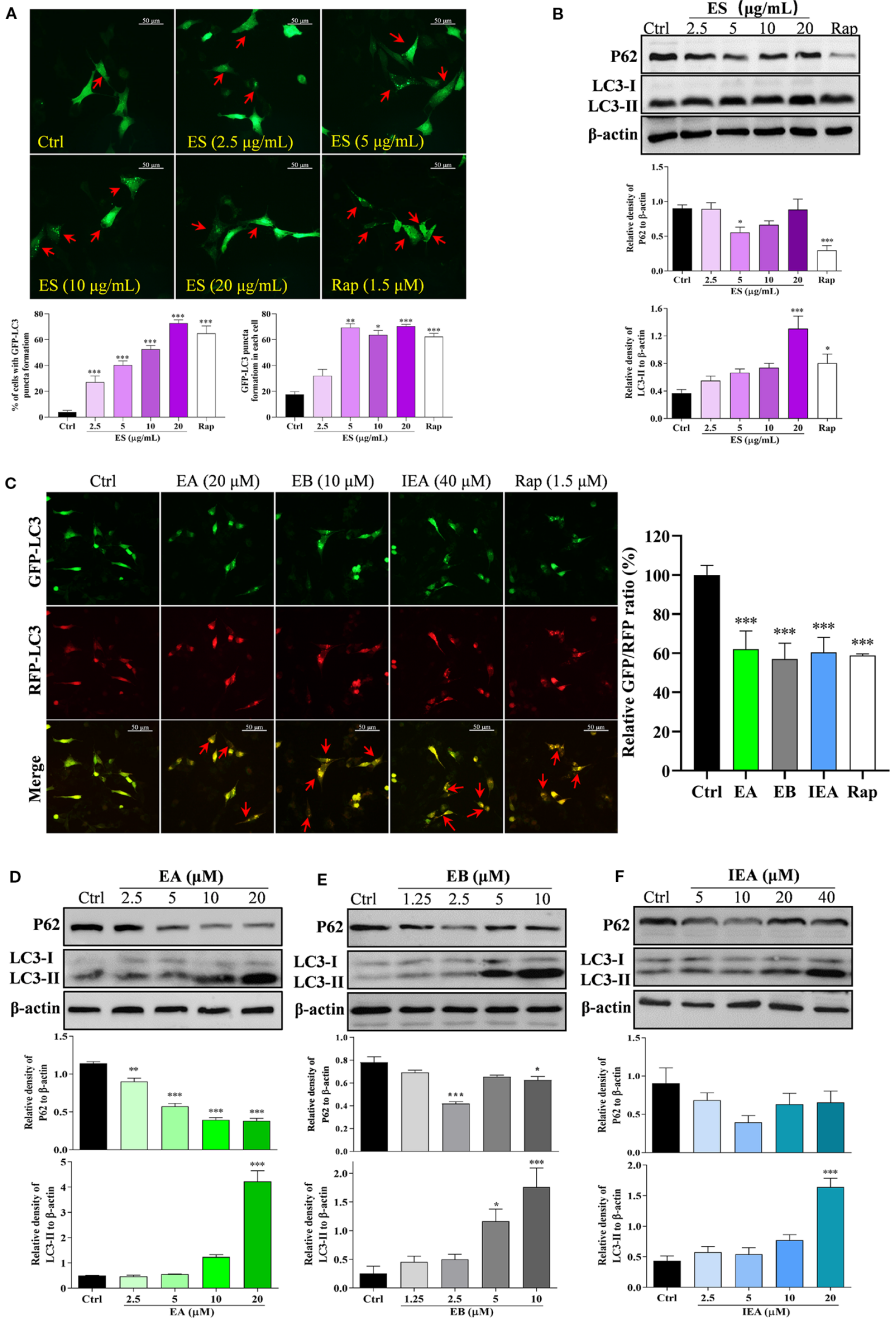
ES, EA, EB, and IEA induce autophagy in HT22 cells. **(A)** HT22 cells transfected with GFP-LC3 were treated with ES and Rap under the indicated concentrations for 24 h. After treatment, the cells were then fixed and the representative images were captured by fluorescence microscope. Red arrows indicate the cells with formed GFP-LC3 puncta. Magnification: 40×. Scale bar: 50 μm. Bar chart indicates the percentage of cells with GFP-LC3 puncta formation and the number of GFP-LC3 puncta in each cell; bars, S.D. *P ≤ 0.05, **P ≤ 0.01, ***P ≤ 0.001. **(B)** HT22 cells were treated with ES and Rap under the indicated concentrations for 24 h. Cell proteins were then harvested for the Western blot analysis of P62, LC3 and β-actin. Bar chart indicates the relative expression of P62 and LC3-II to β-actin; bars, S.D. *P ≤ 0.05, ***P ≤ 0.001. **(C)** HT22 cells transfected with tfLC3 plasmid were treated with EA, EB and IEA at the indicated concentrations for 24 h. After treatment, cells were then fixed and the representative images were captured by fluorescence microscope. Red arrows indicate the cells with undegraded RFP-LC3 puncta. Magnification: 40×. Scale bar: 50 μm. Bar chart indicates the relative ratio of GFP/RFP; bars, S.D. ***P ≤ 0.001. HT22 cells were treated with EA **(D)**, EB **(E)**, and IEA **(F)** at the indicated concentrations for 24 h. Cell proteins were then harvested for the Western blot analysis of P62, LC3 and β-actin. Bar chart indicating the relative expression of P62 and LC3-II to β-actin; bars, S.D. *P ≤ 0.05, ***P ≤ 0.001. The full-length Western blot images are showed in **Figure S13**.

A growing amount of evidence indicates that autophagy plays an important role in degrading the mHtt associated with HD. Therefore, in this study, we investigated whether ES, EA, EB, and IEA could activate autophagy in HT22 cells. In accordance with previous studies (Fleming et al., 2011; Martin et al., 2015; Lee and Kim, 2019), LC3, a specific marker protein, plays an important role in monitoring autophagy. In addition, P62, an autophagy substrate, acts as a reporter of autophagy activity (Galluzzi et al., 2017; Ravanan et al., 2017; Wu et al., 2018). After autophagy induction, intracellular LC3-I is primarily converted into LC3-II and then accumulates on the autophagosome membrane, which is accompanied by the downregulation of P62 protein expression. In addition, the formed enhanced green fluorescent protein fused microtubule-associated protein light chain 3 (EGFP-LC3) puncta dispersing uniformly in the cytoplasm can be observed. Therefore, in the current study, we monitored autophagy primarily by observing GFP-LC3 puncta formation and measuring the expression levels of the protein conversion of LC3-II, and protein expression of P62. By transiently transfecting the pEGFP-LC3 plasmid into HT22 cells, ES enhanced GFP-LC3 puncta formation in a dose-dependent manner (**Figure 4A**). As revealed by Western blot analysis, the protein expression of LC3-II was also increased in a dose-dependent manner, and P62 was correspondingly decreased by ES (**Figure 4B**). Because EA, EB, and IEA are the main components of ES, we herein investigated and validated the effect of EA, EB, and IEA on the autophagy activation in HT22 cells. As shown in **Figure S10**, EA, EB, and IEA indeed dose-dependently increased GFP-LC3 puncta formation in the transiently transfected pEGFP-LC3 plasmid HT22 cells. Furthermore, the autophagic flux of cells was monitored by calculating the GFP/RFP fluorescence ratio in tfLC3 transiently transfected HT22 cells and stable RFP-GFP-LC3 U87 cells. As shown in **Figure 4C** and **Figure S11**, EA, EB, IEA, and Rap significantly decreased the GFP/RFP fluorescence ratio in both HT22 (**Figure 4C** and **Figure S9**) and U87 cells, respectively. Consistent with the results from fluorescence microscopy, EA, EB, and IEA also dramatically increased the expression of the LC3-II protein and decreased the levels of P62 protein in a dose-dependent manner (**Figures 4D–F**). Thus, these results reveal that EA, EB, and IEA are the bioactive compounds in ACB that induce autophagy flux in HT22 cells.

### EB Induces Autophagy Through the mTOR and ERK Signaling Pathways

**Figure 5:**
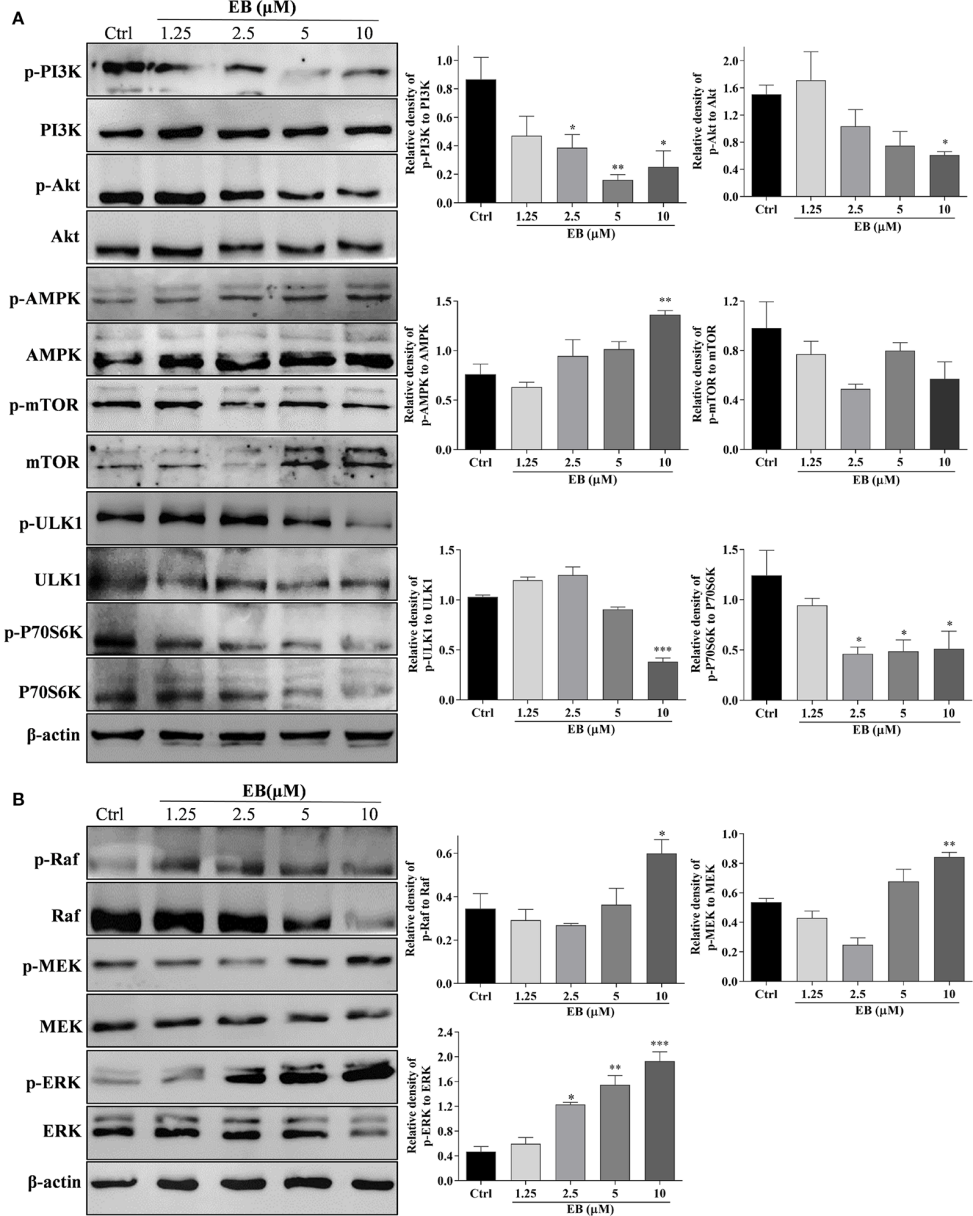
EB regulates both the mTOR and ERK signaling pathways. HT22 cells were treated with EB at the indicated concentrations for 24 h. Cell proteins were then harvested for the Western blot analysis of p-PI3K, PI3K, p-Akt, Akt, p-AMPK, AMPK, p-mTOR, mTOR, p-ULK1, ULK1, p-P70S6K, P70S6K, and β-actin **(A)**, and p-Raf, Raf, p-MEK, MEK, p-ERK, ERK, and β-actin **(B)**. Bar chart indicates the relative expression of phosphorylation protein to total protein; bars, S.D. *P ≤ 0.05, **P ≤ 0.01, ***P ≤ 0.001. The full-length Western blot images are showed in **Figure S14**.

The mTOR (mammalian target of rapamycin) kinase plays a critical role in cell growth and proliferation (Cargnello et al., 2015; Han et al., 2015). As well known, mTOR is a central regulator that is suppressed in response to cellular conditions, such as starvation (Chen et al., 2014; Lastres-Becker et al., 2016), growth factor deprivation and stress, which leads to the activation of autophagy (Zhao et al., 2017). mTOR is negatively regulated by AMPK and positively regulated through the PI3K/Akt pathway (Heras-Sandoval et al., 2014; Qin et al., 2017; Zheng et al., 2017; Zhang et al., 2017; Wang et al., 2018; Rai et al., 2019). The downstream targets of mTOR, such as ULK1 and P70S6K, are inhibited as autophagy is activated (Heras-Sandoval et al., 2014; Abd-Elrahman and Ferguson, 2019). In this study, because EB with similar structure as EA and IEA exhibits potent ability to induce autophagy and degrade mHtt, we selected EB for further investigation of the mechanisms that modulate autophagy induction in HT22 cells. As shown in **Figure 5A**, EB could significantly suppress the PI3K/Akt pathway and increase the activity of AMPK, which then inhibit mTOR activity. Correspondingly, downstream proteins such as ULK1 and P70S6K were inhibited. Therefore, mTOR plays an important role in EB-induced autophagy in HT22 cells. In addition to the classic mTOR signaling pathway, extracellular signal-regulated kinase (ERK1/2) signaling, which promotes cellular proliferation in response to growth factors, can also upregulate the expression of autophagic and lysosomal genes (Bi et al., 2017; Kim et al., 2017; Rai et al., 2019). Therefore, we also investigated the modulation of the MEK/ERK pathway in EB-induced autophagy in HT22 cells. As shown in **Figure 5B**, EB could significantly increase the phosphorylation of Raf, MEK and ERK, suggesting that EB could activate the Raf/MEK/ERK signaling pathway.

**Figure 6:**
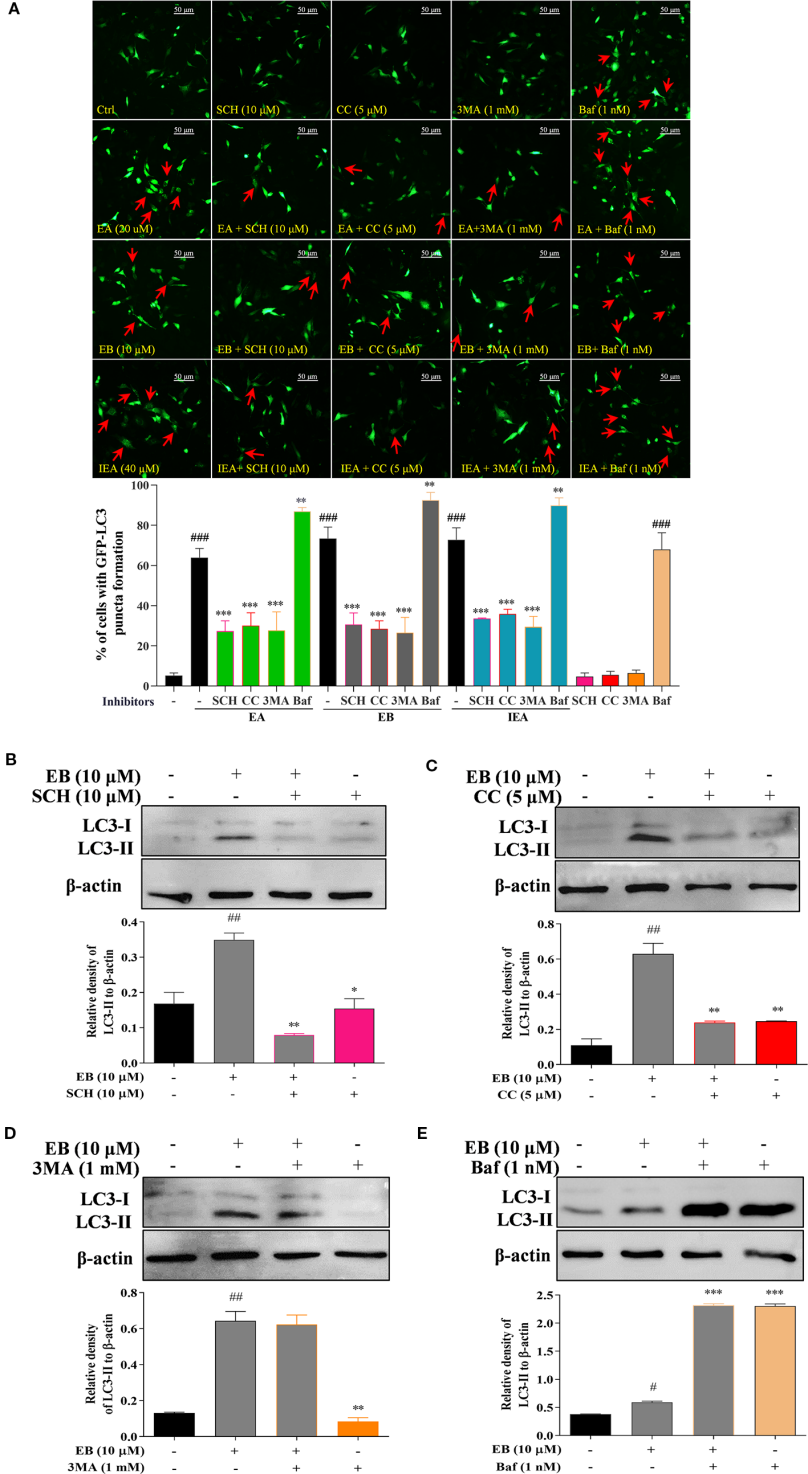
EB activates autophagy *via* both the mTOR and ERK signaling pathways. **(A)** HT22 cells transfected with GFP-LC3 were treated with EB or co-treated with EB and the inhibitors, such as SCH, CC, 3MA, and Baf, at the indicated concentrations for 24 h. After treatment, cells were fixed and the representative images were captured by fluorescence microscope. Red arrows indicate the cells with formed GFP-LC3 puncta. Magnification: 40×. Scale bar: 50 μm. Bar chart indicates the percentage of cells with GFP-LC3 puncta formation; bars, S.D. HT22 cells were treated with EB or co-treated with the inhibitors, such as SCH **(B)**, CC **(C)**, 3MA **(D)**, and Baf **(E)** for 24 h. Cell proteins were then harvested for the Western blot analysis of LC3 and β-actin. Bar chart indicates the relative expression of LC3-II to β-actin; bars, S.D. *^#^P ≤* 0.05, *^##^P ≤* 0.01, *^###^P ≤* 0.001 VS. the control group. ^*^P ≤ 0.05, **P ≤ 0.01, ***P ≤ 0.001 VS. EA, EB, or IEA alone group. The full-length Western blot images are showed in **Figure S15**.

To further demonstrate whether the above signaling pathways are correlated with autophagy activation in EB treated HT22 cells, the specific inhibitors of AMPK and ERK, such as CC and SCH, and the specific autophagy inhibitors including 3MA and Baf that inhibit the initiation and degradation process of autophagy, respectively, were used in the present study. As shown in **Figure 6A**, the percentage of cells with EB-induced formation of GFP-LC3 puncta was significantly inhibited by SCH, CC, and 3MA. However, Baf further increased the percentage of cells with GFP-LC3 puncta formation by impairing the function of lysosome and disrupting autophagic flux (**Figure 6A**). Accordingly, by detecting LC3-II protein expression using Western blot, SCH, CC, and 3MA could significantly decrease the LC3-II expression increased by EB, while Baf further increased the accumulation of LC3-II expression (**Figures 6B–E**). Taken together, the results indicate that the saponins, including EA, EB, IEA, etc., in ACB sharing a similar structure, induce autophagy *via* the mTOR and ERK signaling pathways in HT22 cells.

### EB Enhances the Clearance of mHtt *via* the mTOR and ERK Signaling Pathways Mediated Autophagy

**Figure 7:**
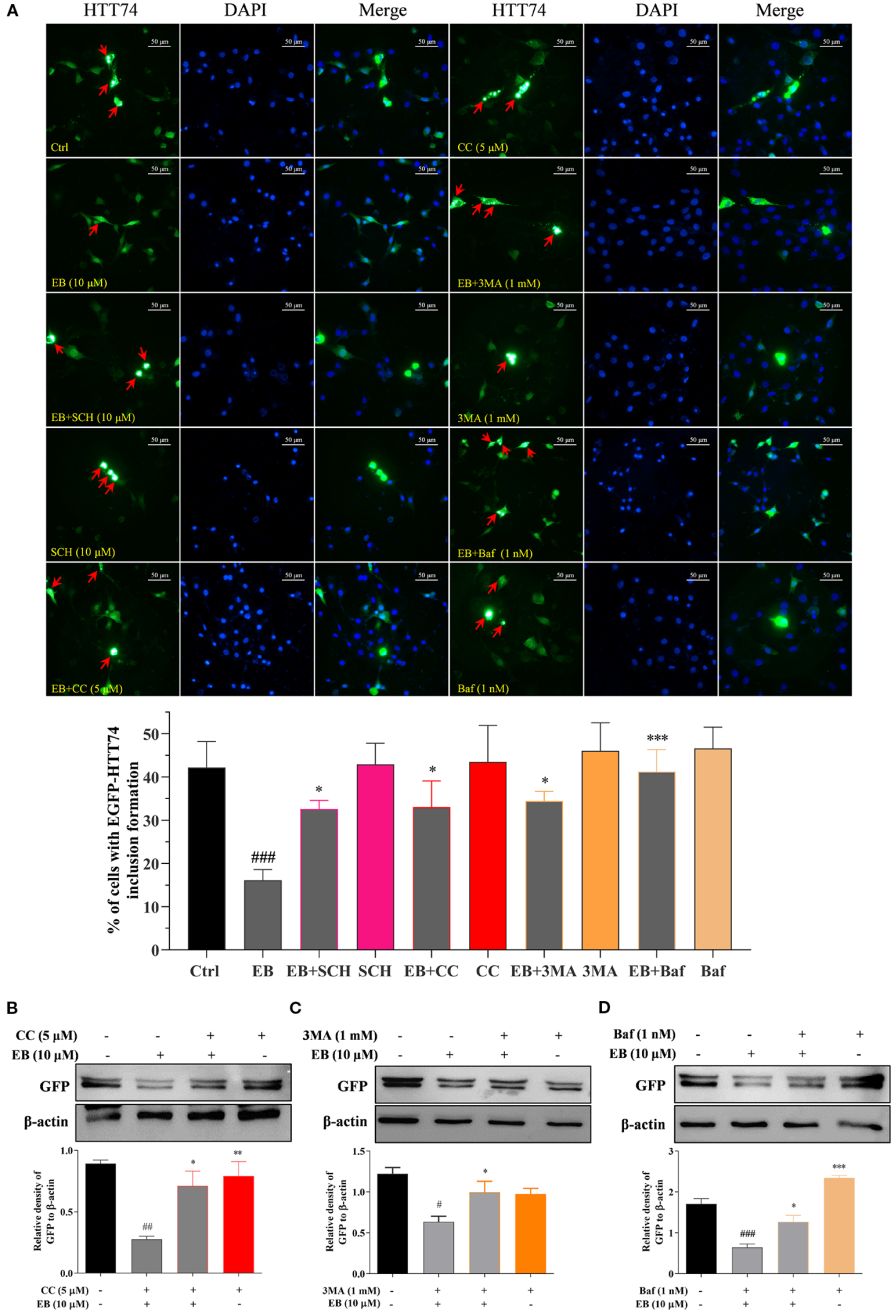
EB enhances the autophagic degradation of mHtt *via* both the mTOR and ERK signaling pathways. **(A)** HT22 cells transfected with EGFP-HTT74 were treated with EB or co-treated with EB and the inhibitors, such as SCH, CC, 3MA and Baf at the indicated concentrations for 24 h. After treatment, cells were fixed and stained with DAPI solution. Representative images of cells with EGFP-HTT74 inclusions formation were captured by fluorescence microscope. Magnification: 40×. Scale bar: 50 μm. Bar chart indicates the percentage of cells with EGFP-HTT74 inclusion formation. **(B)** HT22 cells transfected with EGFP-HTT74 were treated with EB or co-treated with the inhibitors, such as CC **(B)**, 3MA **(C)** and Baf **(D)** at the indicated concentrations for 24 h. Cell proteins were then harvested for the Western blot analysis of GFP and β-actin. Bar chart indicates the relative expression of GFP to β-actin; bars, S.D. *^#^P* ≤ 0.05, *^##^P* ≤ 0.01, *^###^P* ≤ 0.001 VS. the control group. ^*^P ≤ 0.05, **P ≤ 0.01, ***P ≤ 0.001 VS. EA, EB or IEA group. The full-length Western blot images are showed in **Figure S16**.

In this study, to further demonstrate that autophagy induced by EA, EB and IEA is closely linked with the degradation effect of mHtt in the transiently transfected EGFP-HTT74 HT22 cells, the autophagy inhibitors such as 3MA and Baf, and the ERK and AMPK inhibitors including SCH and CC were used. As shown in the fluorescence images presented in **Figure 7A**, 3MA, Baf, SCH, and CC could significantly suppress the degradation effect of EB to EGFP-HTT74 inclusion. Additionally, the Western blot results showed that CC (**Figure 7B**), 3MA (**Figure 7C**) and Baf (**Figure 7D**) could reversed the protein level of GFP which was inhibited by EB. Therefore, the above results indicate that EA, EB, and IEA, etc., in ACB degrade mHtt *via* the ERK and mTOR signaling pathways mediated autophagy.

### EB Enhances the Clearance of mHtt *via* ATG7

**Figure 8:**
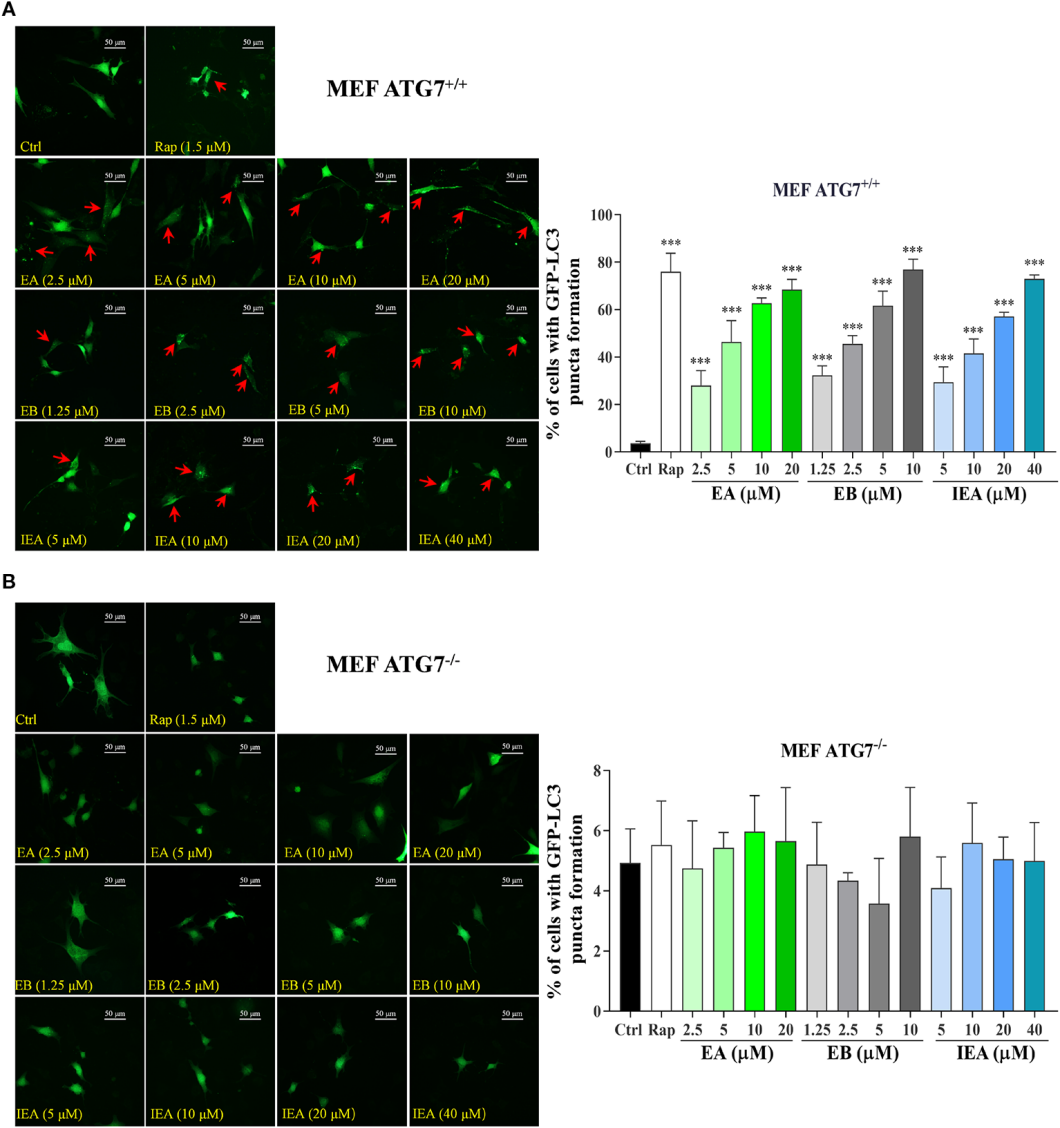
Effects of EA, EB, and IEA on GFP-LC3 puncta formation in ATG7^+/+^ MEF and ATG7^–/–^ MEF cells. ATG7^+/+^ MEF cells **(A)** and ATG7^–/–^ MEF cells **(B)** cells transfected with GFP-LC3 were treated with Rap, EA, EB, and IEA at the indicated concentrations for 24 h. After treatment, the cells were fixed and the representative images were captured by fluorescence microscope. Red arrows indicate the cells with formed GFP-LC3 puncta. Magnification: 40×. Scale bar: 50 μm. Bar chart indicates the percentage of cells with GFP-LC3 puncta formation; bars, S.D. ***P ≤ 0.001.

**Figure 9:**
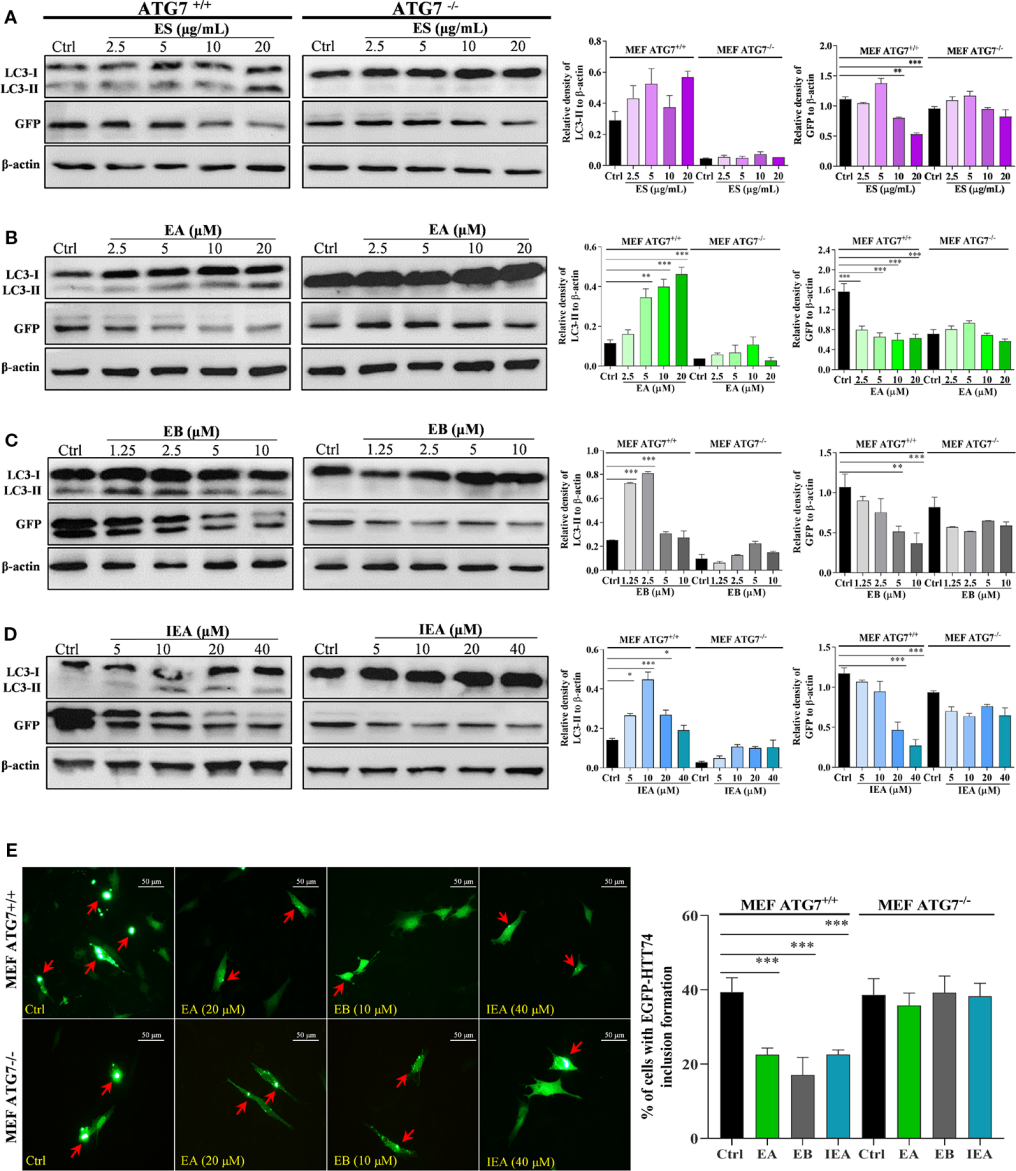
EA, EB, and IEA enhance the clearance of mHtt *via* ATG7. ATG7^+/+^ MEF and ATG7^–/–^ MEF cells transfected with EGFP-HTT74 were treated with ES **(A)**, EA **(B)**, EB **(C)** and IEA **(D)** at the indicated concentrations for 24 h. After treatment, cell proteins were then harvested for the Western blot analysis of LC3, GFP and β-actin. Bar chart indicates the relative expression of LC3-II and GFP to β-actin; bars, S.D. *P ≤ 0.05, **P ≤ 0.01, ***P ≤ 0.001. The full-length Western blot images are showed in **Figure S17**. **(E)** ATG7^+/+^ MEF and ATG7^–/–^ MEF cells transfected with EGFP-HTT74 were treated with EA, EB and IEA at the indicated concentrations for 24 h. After treatment, the cells were fixed and the representative images were captured by fluorescence microscope. Red arrows indicate the cells with formed EGFP-HTT74 inclusion. Magnification: 40×. Scale bar: 50 μm. Bar chart indicates the percentage of cells with EGFP-HTT74 inclusion formation; bars, S.D. ***P ≤ 0.001. The full-length Western blot images are showed in **Figure S17**.

Autophagy-related genes (ATGs), including ATG5 and ATG7, are believed to be the key molecules that are essential for autophagy induction (Leidal et al., 2018; Lee and Kim, 2019). Among them, ATG7 as an E1-like enzyme is required for the conjugation of ATG12 to ATG5, and the conjugation of phosphatidylethanolamine (PE) to LC3-I is mediated by the actions of ATG3 and the ATG5-ATG12 complex. Then, LC3-I is translocated from the cytosol to the isolation membrane and becomes membrane-bound LC3-II (Lee and Kim, 2019). Therefore, to further investigate whether EB enhances the clearance of mHtt *via* ATG7-dependent autophagy, wild-type (ATG7^+/+^ MEF) and ATG7-deficient MEF cells (ATG7^–/–^ MEF) were used. The autophagic activation of MEF cells upon the treatment with EA, EB and IEA was first investigated. As shown in **Figure 8**, EA, EB, and IEA significantly enhanced GFP-LC3 puncta formation in ATG7^+/+^ MEF cells (**Figure 8A**) but not in ATG7^–/–^ MEF cells (**Figure 8B**). Furthermore, the Western blot results showed that ES, EA, EB, and IEA could increase the protein level of LC3-II in a dose-dependent manner and correspondingly decrease the protein level of mHtt in ATG7^+/+^ MEF cells but not in ATG7^–/–^ MEF cells, which were transiently transfected with EGFP-HTT74 (**Figures 9A–D**). As shown in the fluorescence image in **Figure 9E**, EA, EB, and IEA decreased the formation of EGFP-HTT74 inclusions in the ATG7^+/+^ MEF cells, but there was no significant reduction observed in the ATG7^–/–^ MEF cells. Taken together, these results reveal that ATG7 is a key molecule for the activation of autophagy and is essential for the autophagic degradation of mHtt by EB.

### EA, EB, and IEA Decrease the Cytotoxicity of mHtt in HT22 Cells

**Figure 10:**
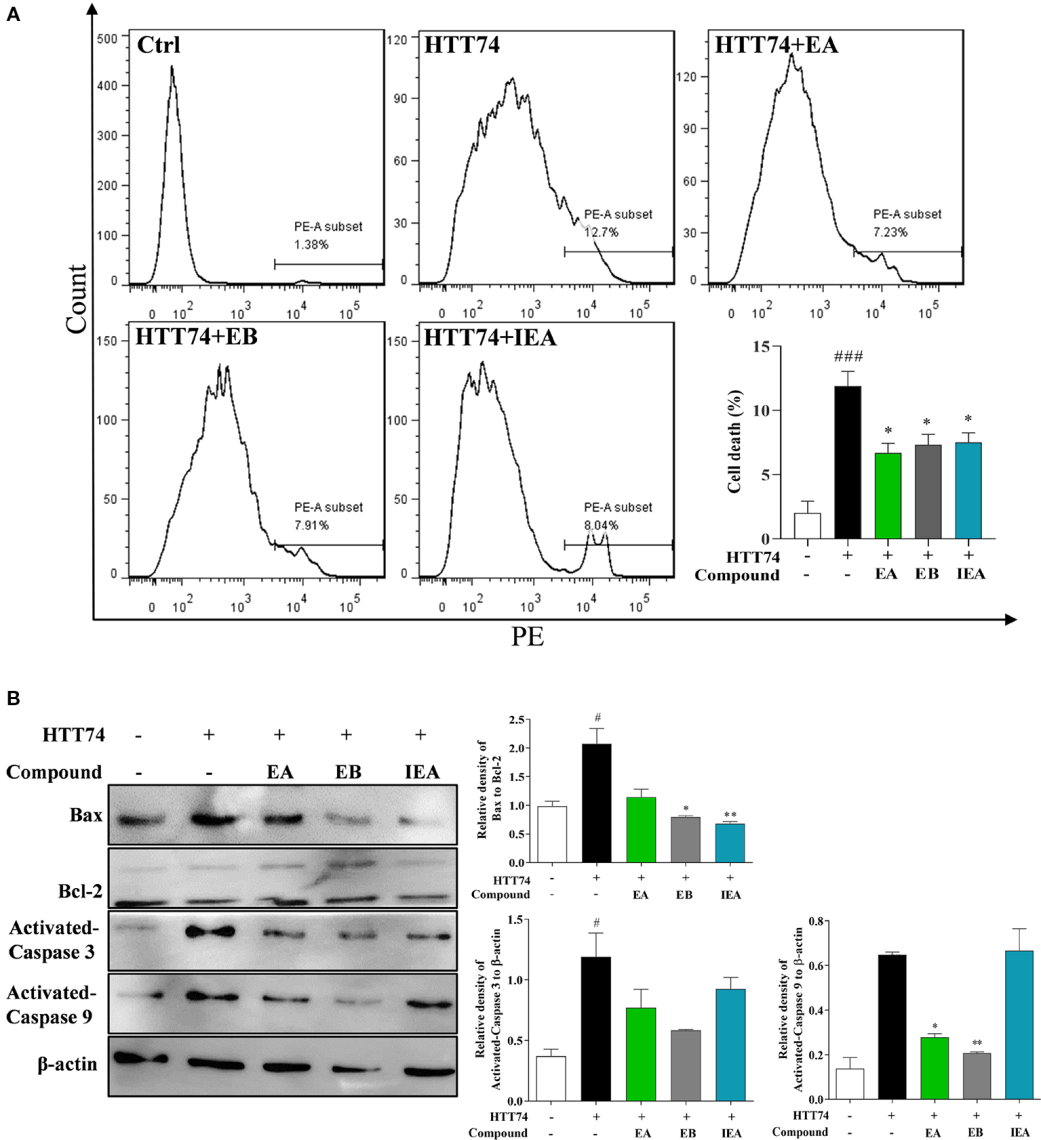
EA, EB, and IEA decrease mHtt-induced cytotoxicity in HT22 cells. **(A)** HT22 cells transfected with EGFP-HTT74 were treated with EA, EB and IEA at the indicated concentrations for 24 h. After treatment, cells were trypsinzed and stained with PI solution for 15 min, then the cells were analyzed by ﬂow cytometry using the PE channel. Bar chart indicates the percentage of dead HT22 cells; bars, S.D. *^###^P* ≤ 0.001 VS. the control group. *P ≤ 0.05, VS. the EGFP-HTT74 group. **(B)** HT22 cells transfected with EGFP-HTT74 were treated with EA, EB and IEA at the indicated concentrations for 24 h. After treatment, cell proteins were harvested for the analysis of Bax, Bcl-2, activated caspase 3, activated caspase 9, and β-actin. Bar chart indicates the relative expression of Bax to Bcl-2, activated caspase 3, and activated caspase 9 to β-actin; bars, S.D. *^#^P* ≤ 0.05 VS. the control group. *P ≤ 0.05, **P ≤ 0.01 VS. the EGFP-HTT74 group. The full-length Western blot images are showed in **Figure S18**.

Intracellular accumulated mHtt is reported to confer the cytotoxicity to neurons (Dayalu and Albin, 2015). In addition to the degradation effect of EA, EB, and IEA to mHtt in HT22 cells, we further investigated the decrease effect of EA, EB, and IEA to mHtt-induced cytotoxicity in HT22 cells. By using flow cytometry analysis together with PI staining, the cell death of HT22 cells was measured by detecting the PI signal. As shown in **Figure 10A**, the PE signal was increased in EGFP-HTT74-overexpressed HT22 cells, while EA, EB, and IEA significantly decreased the corresponding PE signal, suggesting that EA, EB, and IEA could recover the cell viability of EGFP-HTT74-overexpressed HT22 cells. Accumulating evidences indicate that mHtt induces cytotoxicity *via* enhancing oxidative stress and results in the activation of apoptotic signaling pathway (Nellemann et al., 2000; Pandey and Rajamma, 2018). The Bcl-2 family, which includes Bax and Bcl-2, plays a central role in the regulation of the mitochondria-mediated apoptosis (Wu et al., 2016; Mirakhor et al., 2018). Caspase 3 and caspase 9, downstream of the apoptotic signaling pathway, are proteolytic enzymes that function as key proteins to promote apoptosis (Wu et al., 2012a; Yao et al., 2017). In this study, we investigated the effect of EA, EB, and IEA in the expression of apoptosis-related proteins. As shown in **Figure 10B**, the protein levels of Bax/Bcl-2, activated caspase 3 and activated caspase 9 were increased in EGFP-HTT74-overexpressed HT22 cells, while EA, EB, and IEA could significantly reduce the expression of these proteins. Therefore, EA, EB, and IEA can recovery viability of EGFP-HTT74-overexpressed HT22 cells by inhibiting mHtt-induced apoptosis.

## Discussion

Emerging evidence indicates that the aggregated proteins deposited in the particular regions of brain are recognized as constituting the common pathological feature of neurodegenerative diseases (Li et al., 2015; Manoharan et al., 2016), including Alzheimer’s disease (AD), Parkinson’s disease (PD), and Huntington’s disease (HD). For HD, mHtt with 36 or more polyQ repeats has been proven to exist in post-mortem HD brain samples and transgenic HD mouse models, and this accumulated mHtt exhibits cytotoxicity to neurons and accelerates the degeneration of brain (Upadhyay et al., 2018). In addition, with 40 or more polyQ repeats, HD emerges with 100% penetrance (Sarkar et al., 2007). Furthermore, the mid-sized aggregates (33S to 84S) are more stable and correlative with toxicity, which indicates that the mid-sized aggregates, such as 33S to 84S, are optimal choices used for the study (Xi et al., 2016). Therefore, HTT74 was widely used to established the HD cellular model (Ravikumar et al., 2004; Sarkar et al., 2007; Wyant et al., 2017). Therefore, the degradation of perinuclear cytoplasmic aggregates and intracellular inclusions of mHtt have been a promising strategy for HD therapy. Autophagy-mediated lysosomal degradation and the ubiquitin-proteasome system are two major pathways that maintain cell homeostasis by degrading damaged organelles, macromolecules and misfolded proteins, such as Aβ, tau, α-synuclein, and mHtt. Autophagy activated in transgenic knock-in mice expressing an artificial mutant form of huntingtin that lacked the polyglutamine repeat reportedly showed that increased expression of critical autophagy genes slowed down the age-dependent accumulation of damage in neurons and enhanced longevity in Drosophila melanogaster (Fleming et al., 2011). Autophagy enhancer-67 was reported to reduce (128Q) hHTT protein levels in the brain of HD Drosophila (Billes et al., 2016). In addition, the expression of TFEB in the striatum of HDQ^175/Q7^ mice degraded mHtt by increasing the activity of autophagy and lysosomes (Vodicka et al., 2016). Therefore, autophagy induction plays an important role in degrading mHtt. In this work, we explored the autophagy-induced degradation effect of ES, EA, EB and IEA *in vitro*. The preliminary detection of mHtt showed that the main components including EA, EB, and IEA could significantly reduce the protein levels of mHtt in EGFP-HTT74-overexpressing HT22 cells. In the subsequent experiments, the evident inhibitory effect of EA, EB, and IEA on mHtt-induced apoptosis was validated. These results further suggested that these compounds degrade the aggregated proteins *via* autophagy induction and offer neuroprotection.

Currently, many symptomatic drugs are prescribed for HD patients, but they only relieve the symptoms by decreasing dopamine accumulations or interrupting the dopaminergic receptors in the brain. For example, tetrabenazine is characterized by its ability to inhibit the vesicular monoamine transporter 2 (VMAT-2) in the central nervous system (CNS) and can prevent serotonin, dopamine, and norepinephrine in the cytoplasm from uptake into presynaptic vesicles (Wyant et al., 2017; Frank et al., 2017). TCMs with few side effects possess multiple components, targets and efficacies. To date, many Chinese herbs have been reported to be effective in HD cellar and animal models. For example, *Gastrodia elata* attenuated mutant huntingtin-induced protein aggregations by morphological control of mitochondria (Huang et al., 2018). In addition, we previously reported that saponins in *Radix polygalae* and *Hedera helix* and neferine in *Nelumbo nucifera* attenuate the protein levels and toxicity of mHtt in PC-12 cells *via* induction of autophagy through the AMPK/mTOR-dependent pathway (Vincent et al., 2015; Wu et al., 2017; Chen et al., 2018). However, there are no any autophagy enhancers with degradation effects on mHtt have been developed as novel drugs to use in clinical practice, and therefore the further identification of autophagy inducers that degrade mHtt is essential. In this study, ES, the total triterpenoid saponin and its derived components including EA, EB, and IEA isolated from the ACB seeds, was found to induce autophagy. Among them, EB activated autophagy with lower concentration than EA and IEA in HT22 cells. The results in **Figure 1** indicated that EA, EB, and IEA with same molecular formulas have little difference in the structure. The red labeled part of structures displayed that the site of chemical groups, such as hydroxy and ester, and the configuration of olefinic bond decided the autophagy induction effect of these saponins.

mTOR as the classic signaling pathway plays an important role in the regulation of autophagy. The upstream targets, including PI3K/AKT and AMPK, can positively and negatively mediate the activity of mTOR. In the present study, EA, EB and IEA isolated from the ES can suppress PI3K/AKT and activate AMPK, which then inhibit the activity of mTOR and downregulates P70S6K and ULK1. In addition to the mTOR signaling pathway, EA, EB and IEA were found to activate the ERK signaling pathway. Furthermore, CC and SCH, specific inhibitors of AMPK and ERK, respectively, significantly inhibited the expression levels of LC3-II and decrease the formation of GFP-LC3 puncta. In addition, ATG7, an autophagy-related gene, plays a critical role in autophagy induction and the progression of neurodegenerative disease. In this study, by using wild-type and ATG7-deficient MEF cells, we confirmed that the effect of ES, EA, EB, and IEA on autophagy in HT22 cells was ATG7-dependent. Taken together, these results suggest that the saponins in ACB, such as EA, EB, and IEA, activate autophagy *via* ATG7 and both the mTOR and ERK signaling pathways.

**Figure 11:**
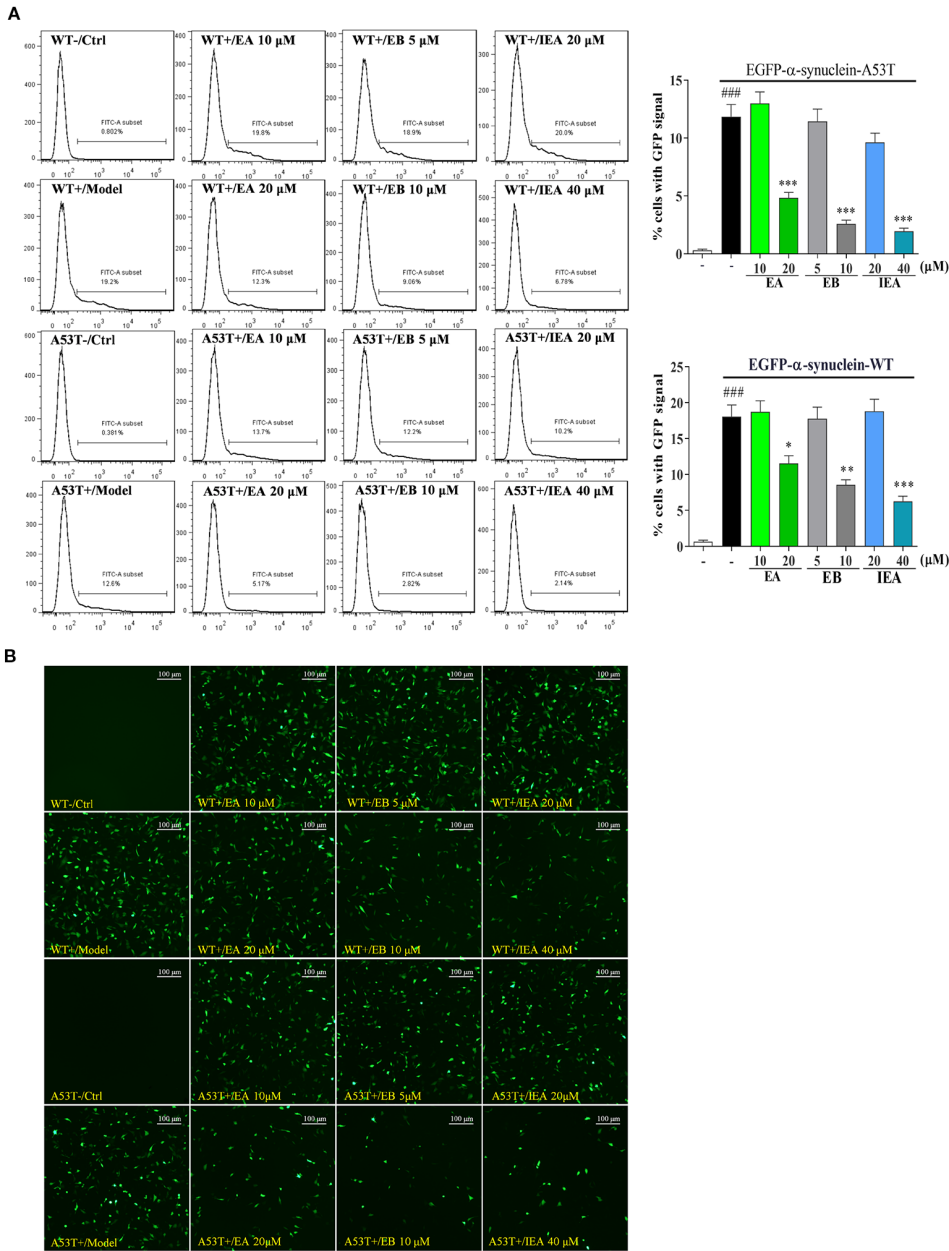
EA, EB, and IEA inhibit the expression of α-synuclein. **(A)** HT22 cells transfected with EGFP-WT-α-synuclein or EGFP-A53T-α-synuclein were treated with EA, EB, and IEA at the indicated concentrations for 24 h. After treatment, cells were trypsinzed and analyzed by ﬂow cytometry using the FITC channel. Bar chart indicates the percentage of cells with a GFP signal; bars, S.D. *^###^P ≤* 0.001 VS. the control group. ^*^P ≤ 0.05, **P ≤ 0.01, ***P ≤ 0.001 VS. the EGFP-HTT74 group. **(B)** HT22 cells transfected with EGFP-WT-α-synuclein or EGFP-A53T-α-synuclein were treated with EA, EB and IEA at the indicated concentrations for 24 h. After treatment, cells were fixed and the representative images were captured by fluorescence microscope. Magnification: 20×. Scale bar: 100 μm.

ES, the total saponin extracted from the seed of ACB, was reported to have anti-inflammatory, anti-oedema, anticancer, hyperglycaemic, anti-obesity, and anti-ulcerative effects (Sirtori, 2001; Zhang et al., 2010; Zhu et al., 2017). In addition, ES can mitigate the chronic MPTP/p-induced dopaminergic toxicity by attenuating mitochondrial dysfunction, oxidative stress, and apoptosis. However, the bioactive components in ES, and the effect and molecular mechanism in PD and HD remain unknown. In this study, through isolation and identification, three saponins including EA, EB and IEA were obtained and confirmed in ES. By overexpressing EGFP-HTT74 in HT22 cells, these saponin could degrade mHtt and decrease its induced cytotoxicity in HT22 cells. Further study demonstrated that the degradation effect of EA, EB, and IEA on mHtt is closely associated with the autophagy induction. In addition to mHtt, the degradation effect of EA, EB, and IEA on PD associated protein α-synuclein was also investigated. As shown in **Figure 11**, EA, EB, and IEA significantly decreased the percentage of cells with GFP signal in transiently transfected EGFP-WT-α-synuclein or EGFP-A53T-α-synuclein HT22 cells. Taken together, these findings show that the saponins in ACB exert neuroprotective effect by accelerating the clearance of misfolded proteins, such as mHtt and A53T-α-synuclein, *via* autophagy. Therefore, the findings of this study are the first to identify the autophagy enhancers from ACB and their ability to clear misfolded proteins, suggesting that the saponins in ACB, including EA, EB, and IEA, are valuable for further study *in vivo* and may be novel candidates for development of therapies against neurodegenerative diseases such as HD and PD.

## Conclusions

In conclusion, this is the first study to demonstrate that the escins, including EA, EB, and IEA in ACB are able to enhance the clearance of mHtt and A53T α-synuclein in HD and PD associated *in vitro* models. Moreover, these saponins can induce potent autophagy in HT22 cells. Among these saponins, EB has a similar structure to other saponins and activates the strongest autophagy *via* ATG7 and both the mTOR and ERK signaling pathways. Furthermore, the degradation effect of these saponins on mHtt has been proven to be closely linked with the autophagy induction. In addition, mHtt-induced apoptosis of HT22 cells can be suppressed by EA, EB, and IEA. Therefore, the findings in this study provide information on the novel pharmacological effects of EA, EB, and IEA in ACB, which are valuable for further study *in vivo* and for development into new candidates for the treatment of neurodegenerative diseases, such as HD and PD, in the future.

## Data Availability Statement

All datasets generated for this study are included in the article/**Supplementary Material**.

## Author Contributions

AW and JW conceived and designed the experiments, contributed new reagents and analysis tools, and supervised all the research. YS, XJ, RP, XZ, RX, YW, and QQ performed the experiments. AW, XZ, and YS wrote the original manuscript. XJ, RP, QQ, and RX analyzed the data. DQ, JW, and AW revised the manuscript. All authors approved the final version of the manuscript.

## Funding

This work was supported by the National Natural Science Foundation of China (grant no. 81774013 and 81903829); the Science and Technology Planning Project of Sichuan Province, China (grant no. 2018JY0237, 2018JY0474, 2019YFSY0014 and 2019JDPT0010); the Administration of Traditional Chinese Medicine of Sichuan Province, China (grant no. 2018QN070 and 2018JC013); the Educational Commission of Sichuan Province, China (grant no. 18TD0051) and the Joint Project of Luzhou Municipal People’s Government and Southwest Medical University, China (grant no. 2016LZXNYD-T03, 2019LZXNYDJ05, 2018LZXNYD-ZK41, and 2018LZXNYD-YL05).

## Conflict of Interest

The authors declare that the research was conducted in the absence of any commercial or financial relationships that could be construed as a potential conflict of interest.
