# Supplementary material for: Escins Isolated from Aesculus chinensis Bge. Promote the Autophagic Degradation of Mutant Huntingtin and Inhibit its Induced Apoptosis in HT22 cells
Source: Front Pharmacol. 2020 Feb 25;11:116. doi: 10.3389/fphar.2020.00116 (PMC7052340; doi:10.3389/fphar.2020.00116)
Supplement: Supplementary file 1 [file DataSheet_1.pdf]

Supplementary Material

1 Supplementary Data

Supplementary Material should be uploaded separately on submission. Please include any supplementary data, figures and/or tables. All supplementary files are deposited to FigShare for permanent storage and receive a DOI.

Supplementary material is not typeset so please ensure that all information is clearly presented, the appropriate caption is included in the file and not in the manuscript, and that the style conforms to the rest of the article. To avoid discrepancies between the published article and the supplementary material, please do not add the title, author list, affiliations or correspondence in the supplementary files.

2 Supplementary Figures and Tables

For more information on Supplementary Material and for details on the different file types accepted, please see here. Figures, tables, and images will be published under a Creative Commons CC-BY licence and permission must be obtained for use of copyrighted material from other sources (including re-published/adapted/modified/partial figures and images from the internet). It is the responsibility of the authors to acquire the licenses, to follow any citation instructions requested by third-party rights holders, and cover any supplementary charges.

2.1 Supplementary Figures

Supplementary Figure

Figure A1. The TIC and DAD chromatogram with UV at 224 nm of the ethanol extract of ACB

Figure A2. The ^{1}H-NMR spectrum of EA

Figure A3. The ^{13}C-NMR spectrum of EA

Figure A4. The ^{1}H-NMR spectrum of EB

Figure A5. The ^{13}C-NMR spectrum of EB

Figure A6. The ^{1}H-NMR spectrum of IEA

Figure A7. The ^{13}C-NMR spectrum of IEA

Figure A8. The full-length western blotting images of Fig. 3A-3E

Figure A9. The full-length western blotting images of Fig. 4B, 4D-3F

Figure A10. The full-length western blotting images of Fig. 5A-5B

Figure A11. The full-length western blotting images of Fig. 6B-6E

Figure A12. The full-length western blotting images of Fig. 7B-7D

Figure A13. The full-length western blotting images of Fig. 9A-9D

Figure A14. The full-length western blotting images of Fig. 10B
